# Effects of exercise training on nitric oxide, blood pressure and antioxidant enzymes

**DOI:** 10.3164/jcbn.16-108

**Published:** 2017-04-07

**Authors:** Yorika Tsukiyama, Tatsuo Ito, Kenjiro Nagaoka, Eri Eguchi, Keiki Ogino

**Affiliations:** 1 Department of Public Health, Okayama University, Graduate School of Medicine, Dentistry and Pharmaceutical Sciences, 2-5-1 Shikata-cho, Okayama 700-8558, Japan

**Keywords:** exercise, blood pressure, l-arginine, nitric oxide, intervention research

## Abstract

The relationship between exercise training and nitric oxide-related parameters was examined in a cross-sectional study and an intervention study. A cross-sectional study using 184 employees was conducted to observe the association of exercise habits with serum arginase (ELISA and activity), l-arginine, l-citrulline, l-ornithine, NOx, exhaled nitric oxide, blood pressure, FEV1%, hs-CRP, HDL-cholesterol, IgE, and life style factors. An intervention study was also conducted to evaluate the changes of serum arginase I, nitric oxide-related parameters, and mRNA levels of anti-oxidant enzymes in blood monocytes before and after 1 h of aerobic exercise training per day for a month. Exercise habits were associated with increased arginase activity and a moderate alcohol drinking habit, after adjustment with several covariates. Aerobic exercise training induced a decrease in l-arginine and diastolic blood pressure and induced an increase in NO2^−^ and urea. Moreover, mRNA expression of anti-oxidant enzymes, such as catalase and GPX1, and a life elongation enzyme, SIRT3, were significantly increased after aerobic exercise. The results that aerobic exercise training increased NO generation, reduced blood pressure, and induced anti-oxidant enzymes via SIRT3 suggest that exercise training may be an important factor for the prevention of disease by inducing intrinsic NO and anti-oxidant enzymes.

## Introduction

In recent years, the number of patients with hypertension, dyslipidemia, and lifestyle-related diseases, such as diabetes, are on the increase, and the onset of these diseases is associated with the inheritance of genetic factors, pathogens, toxic substances, external environmental factors such as stressors, and lifestyle factors such as diet, exercise, and drinking. Although lifestyle choices, such as a lack of exercise, westernization of eating habits, drinking, and smoking, may be major factors in cardiovascular disease, there is considerable evidence to show that exercise training reduces many of the related risk factors, including high blood pressure, high cholesterol, obesity, and insulin resistance.^(1)^ A meta-analysis showed that regular exercise reduces mean systolic and diastolic blood pressure.^(2)^ Several mechanisms for the reduction of blood pressure by exercise training have been considered.^(3–5)^ Exercise also stimulates nitric oxide (NO) release from endothelial cells.^(6)^ Despite the obvious benefits of physical training, the main exercise-mediated mechanisms involved in the improvement of vascular function in coronary artery disease have not been clearly identified.^(7)^ However, the mechanical relationship of vascular physiology with NO has been connected to the reduction of blood pressure after exercise training. Physical exercise reduces blood pressure and is now broadly recommended by current American and European hypertension guidelines.^(8,9)^ However, there is also considerable evidence to show the contribution of NO to the regulation of blood pressure after exercise training.^(7,10,11)^ since NO has been discovered to be an endothelial-derived relaxing factor and may play an important role in not only endothelial relaxation but also the prevention of endothelial cell dysfunction.^(12,13)^

On the other hand, there is an intriguing increase in NO production in skeletal muscle in response to physical exercise. NO, produced by nitric oxide synthase 3 (eNOS) in the vascular endothelium and produced by NOS1 (neuronal NOS), NOS2 (inducible NOS), and NOS3 in skeletal muscle, is synthesized from l-arginine.^(14)^ In skeletal muscle, NOS1 and NOS3 are expressed.^(15,16)^ Chronic exercise, 90 min/day for 8 weeks, increases the expression of NOS1 and NOS3 in skeletal muscle.^(17)^ In skeletal muscle, NO interacts with the metabolic enzyme, AMPK.^(18)^ NO and AMPK cooperatively regulate PGC1α.^(19)^ 70–90% of NO is stored in *S*-nitrosothiols, the main source of NO in tissues.^(20)^ Moderate exercise increases NO content through the activation of nuclear factor κB.^(21)^

Arginase, a key enzyme in the urea cycle, is involved in indirect regulation of NO by the consumption of l-arginine, which is a common substrate for NOS. Studies on the induction or activation of arginase have focused on pre-atherosclerotic vascular changes and asthmatic airway inflammation as pathophysiological evidence that the consumption of l-arginine by arginase may lead to the reduction of NO, resulting in the reduced enlargement of endothelial or bronchial smooth muscle-associated vascular damage.^(22)^

Serum arginase levels were evaluated in various diseases such as asthma,^(23–25)^ type-2 diabetes mellitus,^(26)^ cancer,^(27)^ and atherosclerosis^(28)^ by activity assay and the ELISA method. In a healthy population, arginase I was associated with oxidative stress, exhaled nitric oxide, and l-arginine.^(29–31)^

Although there is considerable evidence showing the association of exercise training and NO, there are few reports demonstrating the association of exercise training with arginase and NO-related parameters in cross-sectional and intervention studies. Therefore, in this study, we evaluated the interaction of arginase with exercise training in association with NO-related parameters in healthy individuals who were occupational workers and exercise training instructors.

## Materials and Methods

### Study design

A cross-sectional study on the relationship between arginase or NO-related clinical parameters and exercise training was designed for an industrial company (*n* = 408) in Hiroshima Prefecture in September 2011, and all participants provided written informed consent. The data of 184 of those who had no previous history of asthma, diabetes mellitus, or other serious disease were used for the analysis, and 224 individuals were excluded due to irregular employment and shift work. The ethics committee of Okayama University approved the study (Number 561).

An intervention study for exercise training was performed in 41 individuals who were employees of a sports club or students of International Pacific University in Japan. The 41 individuals provided written informed consent. The survey period was from May to July 2013. It was divided into two groups. The first intervention, with 19 individuals, was performed from May 8 to June 3, 2013, and the second intervention, with 22 individuals, was performed from June 16 from July 15, 2013. Continuous exercise training was performed five times per week for 4 weeks. Blood for analysis was withdrawn before and after intervention. The ethics committee of Okayama University approved the study, and all subjects gave written informed consent. This intervention study was registered with UMIN (UMINO000024457).

### Exercise training

Bicycle exercises were performed using a stationary bicycle with an attached ergometer for one hour a day, five times per week, for 4 weeks. In addition, the exercise intensity was limited to “moderate”, such that the subject’s muscles did not consciously feel “tight”. The exercises were managed by setting a target heart rate relative to the subject’s maximum heart rate, calculated by the following Karvonen formula: “target hear trate = [(220 − age) − resting heart rate] × 0.6 + resting heart rate”. For safety, the exercise intensity was set to 60%. The exercise was carried out under the supervision and guidance of exercise guidance staff.

### Measurement of clinical parameters for whole blood and serum

Ten ml of blood samples were collected one day before of beginning of exercise and one day after the day of final exercise. Venous blood samples were collected after 4 h of fasting. Serum for intervention was collected within 10 min and serum for cross-sectional analysis was collected after standing for 1 h to coagulate the blood. Sera were preserved at −80°C until analysis. Serum LDL-cholesterol was measured using automated XE-2100 (Sysmex, Kobe, Japan) and H7700 (Hitachi High-Technologies, Tokyo, Japan), and high-sensitivity CRP (hs-CRP) was measured by a highly sensitive CRP assay (Behring Latex-Enhanced using the Behring Nephelometer BN-100; Behring Diagnostics, Westwood, MA). Arginase activity and IgE were determined using ELISA kits (Montgomery, TX), and arginase I was determined with ELISA kits previously established.^(31)^ Information on lifestyle factors, including cigarette smoking, exercise habits, and alcohol drinking habits, was obtained using self-reported questionnaires or clinical records.

### Fractional exhaled NO (FeNO) and FEV1%

Fractional exhaled NO (FeNO) was measured using a portable electrochemical analyzer (Niox Mino, Aerocrine AB, Sweden). This device measures FeNO during a 10-s exhalation with a constant flow of 50 ml/s, according to the international recommendations. All measurements were performed in duplicate, all within 10% deviation, and the mean concentration in parts per billion (ppb) was registered.

FEV1% was measured as a pulmonary function test using a spirometer (CHESTGRAPH Jr.; Chest, Tokyo, Japan) according to international recommendations, measuring FEV1 and FVC, and expressed as a percentage of FEV1/FVC.

### Measurement of serum NO_2_^−^ and NOx

Serum NO_2_^−^ is gradually oxidized by hemoglobin to NO_3_^−^ within 10 min. Serum NO_3_^−^ is derived from NO_2_^−^, food intake, and intestinal flora. Therefore, serum NO_2_^−^ before oxidation is important for NO supply in the blood. NO_2_^−^ was determined with a NO analyzer (model-280i NOA with the Purge Vessel; Sievers, Boulder, CO).

NOx (NO_2_^−^ + NO_3_^−^) levels in the serum were determined by reduction of NO_3_^−^ to NO_2_^−^. After reduction of NO_3_^−^ to NO_2_^−^ with nitrate reductase (Sigma-Aldrich, St. Louis, MO) for 30 min at room temperature, NOx was determined with an NO analyzer (model-280i NOA with the Purge Vessel; Sievers). In the NO analyzer, NO_2_^−^ was further reduced to NO in the Purge Vessel containing the reducing agent potassium iodide in acetic acid, and generated NO was transported to the NO analyzer using the ozone-chemiluminescence method.

### Measurement of serum l-arginine, l-citrulline and l-ornithine

Serum l-arginine was measured using an HPLC system (HITACHI, Tokyo, Japan). l-Arginine was eluted from serum and supplemented with 5 µM monomethylarginine (MMA) as an internal standard, using Oasis MCX solid phase-extraction cartridges (Waters, Milford, MA) conditioned with 2 ml of methanol/water/ammonia solution (50:45:5, v/v/v) and phosphate-buffered saline (PBS). Serum samples containing 5 µM MMA were dissolved in PBS and loaded on an equilibrated SPE column. The column was constitutively washed with 0.1 N HCl (2 ml) and methanol (2 ml). The fraction containing l-arginine was eluted with 1 ml of methanol/water/ammonia solution (50:45:5, v/v/v) and dried in a vacuum centrifuge. After the drying process, the residue was reconstituted with water. Serum samples for l-citrulline and l-ornithine were measured using an HPLC system (HITACHI). Serum samples for l-citrulline and l-ornithine containing 10 µM MMA were precipitated with 1.5 M HClO_4_. The samples reconstituted with water for l-arginine or the supernatants for l-citrulline and l-ornithine were mixed with an equal amount of derivatizing agent (5 mg/ml ortho-phthaldialdehyde, 10% methanol, 0.5% 3-mercaptopropionic acid in 200 mM borate buffer, pH 9.5), and the reaction was allowed to occur for 30 min at room temperature. The samples for l-arginine were introduced into the fluorescence HPLC system using a TSKgel ODS-100V column (4.6 × 250 mm, 5 µm, Tosoh, Yamaguchi, Japan) with the mobile phase consisting of 9% acetonitrile in acetate buffer (pH 6.3) at a flow rate of 1.5 ml/min. The samples for l-citrulline and l-ornithine were examined with the fluorescence HPLC system using a Supelco C18 column (4.6 × 15 cm, 3 mm, Bellefonte, PA) at a flow rate of 1.1 ml/min of a gradient mixture as follows: mobile phase A, consisting of 0.1 M sodium acetate (pH 7.2) containing of 9% methanol and 0.5% tetrahydrofuran, to mobile phase B consisting of 100% methanol. The detection of each amino acid was performed using excitation and emission wavelengths of 340 and 455 nm, respectively.

### Expression of mRNA of anti-oxidative enzymes in peripheral blood monocytes in the intervention study

 After intervention, blood with anti-coagulant was put on Polymorphprep (Axis-Shield PoC AS, Oslo, Norway), and a cell layer of monocytes was removed and washed with Hank’s balanced solution. Total RNA was purified from monocytes by ISOGEN (NIPPON GENE, Tokyo, Japan) in combination with High-Salt Precipitation Solution (NIPPON GENE). RNA concentrations were measured on a Nano Drop 1000 spectrophotometer (NanoDrop Technologies, Wilmington, DE). The integrity of RNA was verified by denaturing agarose gel electrophoresis. Complementary DNA (cDNA) was synthesized from 1 µg of total RNA in a 20-µl reaction volume using the PrimeScript 1st strand cDNA Synthesis Kit (Takara6110) with the Oligo dT primer, according to the manufacturer’s instructions. An aliquot of cDNA was used as a template for quantitative PCR using SYBR Premix Ex Taq (Tli RNaseH Plus) (Takara RR420) with the ROX dye passive reference on the StepOne Plus Real-time PCR System (Applied Biosystems; the Central Research Laboratory, Okayama University Medical School) operated in the relative gene expression mode using the ROX dye as a passive reference. The primers used are listed in Table 6. Primers were chosen from the PrimerBank^(32)^ or qPrimerDepot^(33)^, such that each amplicon spanned at least one intron. Relative expression levels were calculated by the ΔΔCt method using the GAPDH gene as an endogenous control for normalization, with the following modification: the mean PCR amplification efficiency for each primer set was calculated by performing PCR in duplicate using undiluted and 4-fold diluted cDNAs as templates. Expression levels in each sample represented the geometric means of duplicate measurements with the dilution rate and amplification efficiency being taken into account. In a few samples in which multiple peaks were observed in the melting temperature curve of one of the duplicate measurements, the expression level of only one of the duplicates with a single peak was represented. The mean of the relative expression level of post-intervention to pre-intervention was presented.

### Statistical analysis

Statistical analyses were performed using GraphPad Prism 5.0c for Mac (GraphPad Software, Inc., San Diego, CA) and PASW Statistics 18 for Mac. The differences in means ± SEM of several clinical parameters were analyzed between sex, age (<44 and ≥45) with the Mann-Whitney *U* test or unpaired *t* test, and the changes of nitric oxide and l-arginine-related parameters according to exercise degree were analyzed by age and sex-adjusted ANCOVA. We performed logistic regression analysis to investigate the association of exercise with arginase and NO-related clinical parameters using log-transformed values. Covariate for adjustment were log-transformed values of age, MBI, arginase I, arginase activity, l-arginine, l-citrulline, l-ornithine, NOx, FeNO, FEV1%, systolic and diastolic blood pressure, and hs-CRP, HDL-cholesterol, IgE and dichotomized scales variable of smoking habits, exercise, and alcohol consumption. All the probability values for the <0.05 were regarded as statistically significant.

## Results

### Characteristics of subjects of cross-sectional study

 The characteristics of 184 healthy individuals are shown in Table 1. Serum levels of arginase I, arginase activity, l-arginine, and l-ornithine, diastolic blood pressure, and the likelihood of exercising, smoking, and alcohol drinking were significantly higher in males compared with females. l-Ornithine, NOx, and blood pressure were higher in the over-47 age group compared with the under-46 age group. When the degree of exercise was divided into three groups (no exercise training, some exercise training each week, and daily exercise training each week), arginase activity and FENO showed significant differences among the three groups after being adjusted for age and sex by ANOVA (Table 2).

### Logistic regression analysis for exercise training habits

Significantly high odds of exercise training are shown in Table 3. A positive association between the degree of exercise and arginase activity, with significant *p* trend, is shown.

### Characteristics of and changes in clinical and NO-related parameters in the intervention study

The characteristics of the 41 intervention individuals are shown in Table 4. l-Arginine was significantly reduced after exercise intervention compared with pre-intervention. These significant reductions were observed independent of sex and age. A significant increase in NO_2_^−^ observed after exercise intervention, compared with pre-intervention, was found in males. Diastolic blood pressure decreased significantly after intervention in the older age group. Urea synthesis by arginase was increased in post-intervention compared with pre-intervention. This increase was found in females and younger aged individuals.

### Changes in serum l-arginine, l-citrulline, and l-ornithine in the intervention study

To observe the metabolism of amino acids relating to NO synthesis by NOS and urea synthesis by arginase, the ratios of l-arginine, l-citrulline, and l-ornithine were calculated and compared between pre-intervention and post-intervention (Table 5). The ratio of l-arginine/l-citrulline + l-ornithine was significantly decreased after intervention. l-Arginine/l-citrulline and l-arginine/l-ornithine were also significantly decreased after intervention.

### Real-time PCR of mRNA of SIRT3 and anti-oxidative enzymes of superoxide dismutase 1 (SOD1), superoxide dismutase 2 (SOD2), catalase, and glutathione peroxidase 1 (GPX1) in the intervention study

**Fig. 1:**
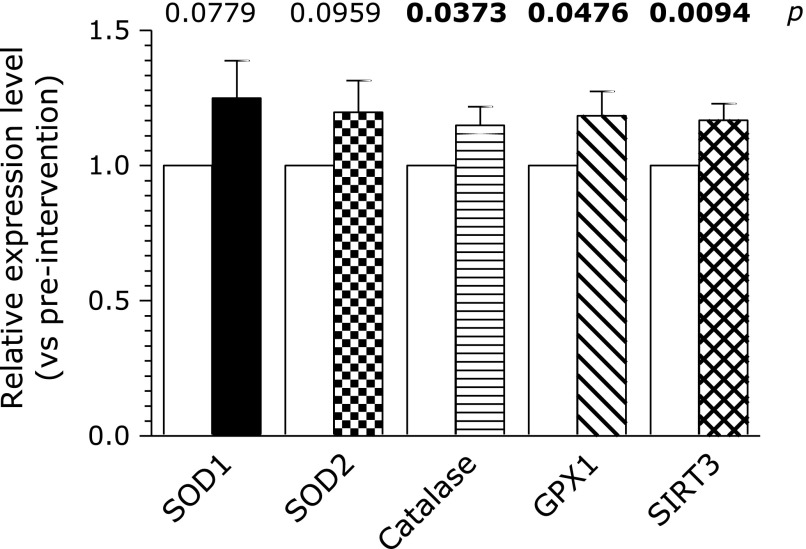
Expression of mRNA of SOD1, SOD2, catalase, GPx1 and SIRT3 in the intervention study of exercise training. Relative mean values of mRNA in post-intervention vs pre-intervention are presented. Bold *p* value represents statistical significance.

The regulation of the mRNA of SIRT3, SOD1, SOD2, catalase, and GPX1 was measured in the intervention study of exercise training by real-time PCR. The messenger RNAs of catalase, GPX1, and SIRT3 were significantly up-regulated in post-exercise compared with pre-exercise (Fig. 1). SOD1 showed a tendency of up-regulation by the intervention of exercise training.

## Discussion

The cross-sectional study with 184 healthy individuals showed the relationship of the degree of exercise with arginase activity and FeNO. In the intervention study, after exercise training, l-arginine was reduced and NO_2_^−^ was increased. Moreover, the ratios of l-arginine/l-citrulline + l-ornithine, l-arginine/l-citrulline, and l-arginine/l-ornithine were significantly reduced. These results suggested that NO production may increase with exercise training. Since the arginase I content in serum was not changed between pre-intervention and post-intervention, and although arginase activity was not measured in the intervention study, it was believed that arginase was modified into a nitrosylated molecule, and activity was increased by NO.^(34)^ The NO supply during exercise contributed to the up-regulation of NOS1 and NOS3 in skeletal muscle.^(16–18)^ Moreover, 70–90% of NO is stored in S-nitrosothiols, the main source of NO in tissues.^(21)^ However, it is not known whether the NO generated in NOS1 and NOS3 in skeletal muscle affects serum NO_2_^−^ levels. NOS3 in vascular endothelial cells has not been ruled out, although there are several controversial studies on exercise-induced changes in NOS3. A 24-week course of swimming did not change the expression of the NOS3 protein in healthy mice.^(35)^ However, exercise is associated with increases in NOS3 expression at the mRNA and protein levels.^(36)^ Moreover, there is considerable evidence to support the idea that the NO supply is regulated by nitrite (NO_2_^−^), and NO_2_^−^ is a physiologically relevant storage reservoir of NO^(37)^ in blood and tissue that can readily be reduced to NO under pathological conditions, such as ischemia or hypoxia.^(38)^ Although NO production is oxygen-dependent in normoxic tissues and is limited in hypoxia, the nitrate-nitrite-NO pathway is significantly facilitated and can complement NOS-based NO production under conditions of exercise or ischemia.^(39)^

Diastolic blood pressure was reduced by intervention in older aged individuals. A reduction of sympathetic tone is reported consistently in different studies and regarded as a mechanism of crucial relevance.^(3,5)^ In the presence of physical exercise, physical and chemical stimuli control NO production.^(11)^ In the endothelial cells, exercise stimulates NO synthesis through chemical mechanisms. These chemical mechanisms involve the interaction of endogenous/exogenous agonists (acetylcholine, bradykinin, and ATP) with the specific receptors on the endothelial cells. In exercise, the efferent nerve neuromuscular junctions are the physiological source of acetylcholine.^(40)^ Interstitial bradykinin content is increased in the contraction during strenuous exercise.^(41)^ Physical impact on the vascular wall stimulates NO release in the blood vessel.^(42)^ Although the mechanisms of shear stress-induced NO synthesis are not completely understood, increased exercise-induced shear stress stimulates the release of a vasorelaxation factor (NO) and augments NOS3 and NOS1 expressions.^(43)^ Moreover, physical activity increases testosterone level in the blood.^(44)^ Testosterone induced NO generation^(45)^ and vascular relaxation via NO generation.^(46)^ Therefore, in this intervention study, elevation of NO_2_^−^ may be involved in the up-regulation of NOS3 activity inducing higher levels of testosterone in vascular endothelial cells.

Serum l-arginine levels, the ratio of l-arginine/l-citrulline + l-ornithine, the ratio of l-arginine/l-citrulline, and the ratio of l-arginine/l-ornithine were reduced by the intervention of exercise training. Arginase activity in the cross-sectional study increased in parallel with the increase in degree of exercise.

*S*-nitrosylation of cysteine residues in the arginase protein molecule by up-regulated NO augment arginase activity.^(33)^ Increased arginase activity may consume l-arginine and up-regulated NOS may consume l-arginine. Therefore, the reduction of l-arginine levels after exercise intervention is understandable.

Physical exercise induced a transient increase in reactive oxygen species (ROS) from mitochondria, and the increased ROS induce the nuclear transcription coactivator, PGC1α/β, and activate several nuclear transcription factors, including PPARγ, and their targets, superoxide dismutase (*SOD*)*1*, *SOD2*, glutathione peroxidase (*GPx*)1, and catalase (*CAT*), resulting in the induction of endogenous ROS defense enzymes in skeletal muscle.^(47)^ Exercise training for 6.5 weeks increased SIRT3 and SOD2 via AMPK activation in skeletal muscle.^(48)^ Moderate exercise induced SOD activity.^(49)^ Moreover, induction up-regulated PGC1α/β, which activated the *SIRT3* gene and resulted in an increase in *GPx1*, *SOD*, and *catalase* via FOXO3α.^(50)^ Therefore, the up-regulation of *GPx1*, *catalase*, and *SIRT3* mRNA in peripheral blood monocytes after exercise training intervention supported the involvement of ROS, PGC1α/β, and AMPK and supported an increase in anti-oxidative capacity. It also suggests that mRNA in peripheral blood monocytes is useful in observing the effect of exercise training, even though most previous studies used the mRNA in skeletal muscle.

Although the findings here are significant, several limitations in the study should be noted. First, the sample size was small. Second, casual relationships could not be determined because one of the studies was a cross-sectional study. Third, this intervention study did not have a control study. Forth, some reporting bias may have been introduced because the information on exercise training was obtained via self-reported questionnaires. Fifth, for an analysis of exercise training, information about clerical staff or physical labor is needed.

The positive association of exercise with arginase activity in the cross-sectional study and decreased l-arginine, decreased diastolic blood pressure, and increased NO_2_^−^ in the intervention study may suggest that exercise training supports the reduction of blood pressure via increase in NO; and the up-regulation of catalase, *GPx1*, and *SIRT3* mRNA suggests exercise training is a prerequisite for the protection of cells from oxidative stress and the support of an elongated life span.

## Figures and Tables

**Table 1:** Characterisitcs of arginase, nitric oxide-related clinical parameters

Variables	Total		Male		Female	*p*	Age <46		Age ≥47	*p*
	(*n* = 184)		(*n* = 120)		(*n* = 64)		(*n* = 92)		(*n* = 92)	
Age	46.3 ± 1.0		45.3 ± 1.3		48.0 ± 1.6	0.225	34.5 ± 0.9		58 ± 0.6	**<0.0001**
Sex (M/F)	120/64						63/29		57/35	0.353
BMI	23.6 ± 0.3		23.9 ± 0.3		22.9 ± 0.4	**0.034**	23.4 ± 0.4		23.7 ± 0.4	0.576
Arginase I	5.2 ± 0.3		5.8 ± 0.3		4.0 ± 0.4	**<0.0001**	5.4 ± 0.4		5.0 ± 0.4	0.292
Arginase activity	4.3 ± 0.2		4.9 ± 0.2		3.2 ± 0.3	**<0.0001**	4.5 ± 0.3		4.2 ± 0.3	0.323
l-Arginine	197.7 ± 3.5		206.7 ± 4.3		181.1 ± 5.4	**0.0005**	195.3 ± 4.7		200.2 ± 5.2	0.604
l-Citrulline	315.6 ± 9.0		322.7 ± 10.6		301.8 ± 16.6	0.372	317.5 ± 11.6		313.7 ± 13.9	0.956
l-Ornithine	345.0 ± 10.1		359.8 ± 13.4		316 ± 13.2	**0.047**	324.7 ± 11.2		366.4 ± 16.6	**0.024**
l-Citru + l-Orni	614.4 ± 16.2		638.3 ± 20.3		578 ± 25.0	**0.037**	607.4 ± 20.5		628.2 ± 24.5	0.631
l-Arg/l-Citru + l-Orni	0.3 ± 0.1		0.3 ± 0.1		0.31 ± 0.1	0.677	0.30 ± 0.1		0.30 ± 0.1	0.203
l-Arg/l-Citru	0.70 ± 0.1		0.68 ± 0.1		0.70 ± 0.1	0.280	0.7 ± 0.1		0.7 ± 0.1	0.570
l-Citru/l-Orni	1.0 ± 0.1		0.9 ± 0.1		1.0 ± 0.1	0.085	1.0 ± 0.1		0.9 ± 0.1	0.073
l-Arg/l-Orni	0.6 ± 0.1		0.6 ± 0.1		0.6 ± 0.1	0.637	0.60 ± 0.1		0.60 ± 0.1	**0.027**
NOx	33.3 ± 1.6		34.9 ± 2.2		30.4 ± 2.0	0.113	29.6 ± 1.4		37.10 ± 2.8	**0.024**
FeNO	20.7 ± 1.4		21.70 ± 1.9		18.7 ± 2.0	0.059	22.5 ± 2.5		18.90 ± 1.3	0.934
FVE1%	88.6 ± 0.6		88.8 ± 0.8		88.4 ± 1.1	0.906	91.8 ± 0.8		85.50 ± 0.9	**<0.0001**
Systolic blood pressure	125.0 ± 1.1		126.2 ± 1.3		122.6 ± 2.2	0.136	120.3 ± 1.4		129.7 ± 1.7	**<0.0001**
Diastolic blood pressure	78.2 ± 0.8		79.9 ± 1.0		74.9 ± 1.4	**0.0027**	75.5 ± 1.1		80.9 ± 1.1	**0.0002**
hs-CRP	0.9 ± 0.1		0.80 ± 0.1		1 ± 0.3	0.102	0.9 ± 0.2		0.80 ± 0.1	**0.015**
HDL-c	57.2 ± 1.2		53.9 ± 1.6		63.4 ± 1.6	**<0.0001**	56.5 ± 2.0		57.90 ± 1.5	0.240
IgE	128.6 ± 12.6		138.5 ± 17.1		110.2 ± 16.9	0.109	151.2 ± 20.8		106.0 ± 14.0	0.065
Exercise habit (+/–)	62/122		50/70		12/52	**0.024**	32/60		30/62	0.756
Smoking habit (+/–)	72/112		60/60		12/52	**<0.0001**	36/57		38/55	0.765
Alcohol drinking (+/–)	108/76		82/38		26/38	**0.0003**	52/40		56/36	0.549

Values represent mean ± SEM, and bold indicates statistical significance.

**Table 2:** Age and sex adjusted mean values of nitric oxide and l-arginine-related clinical variables

	Degree of exercise			*p*
	– (*n* = 119)	+ (*n* = 53)	++ (*n* = 11)	
BMI	23.6 ± 0.4	24.3 ± 0.5	22.6 ± 1.1	0.235
Arginase I	4.9 ± 0.3	5.6 ± 0.5	5.7 ± 1.0	0.432
Arginase activity	3.9 ± 0.2	4.8 ± 0.3	6.3 ± 0.7*	**0.001**
l-Arginine	190.7 ± 4.6	182.9 ± 7.3	161.7 ± 15.1	0.155
l-Citrulline	304.6 ± 10.7	294.9 ± 17.0	266.2 ± 35.0	0.548
l-Ornithine	333.6 ± 12.0	315.9 ± 18.5	285.5 ± 37.9	0.395
l-Citru + l-Orni	615.6 ± 22.8	592.9 ± 33.1	554 ± 68.5	0.625
l-Arg/l-Citru + l-Orni	0.3 ± 0.1	0.3 ± 0.1	0.3 ± 0.1	0.719
l-Arg/l-Citru	0.7 ± 0.1	0.7 ± 0.1	0.6 ± 0.1	0.752
l-Citru/l-Orni	1.0 ± 0.1	0.9 ± 0.1	1.0 ± 0.1	0.593
l-Arg/l-Orni	0.6 ± 0.1	0.6 ± 0.1	0.6 ± 0.1	0.499
NOx	32.3 ± 2.0	34.7 ± 3.1	32.2 ± 6.4	0.904
FeNO	18.6 ± 1.7	20.3 ± 2.7	38.5 ± 5.6*^,^^#^	**0.004**
FVE1%	88.9 ± 0.7	89.1 ± 1.1	86.6 ± 2.3	0.611
Systolic blood pressure	123.9 ± 1.3	127.1 ± 2.1	125.5 ± 4.3	0.418
Diastolic blood pressure	78.4 ± 0.9	77.8 ± 1.5	78.8 ± 3.1	0.920
hs-CRP	0.9 ± 0.1	0.8 ± 0.2	1.7 ± 0.4	0.148
HDL-c	56.4 ± 1.5	58.3 ± 2.3	51.60 ± 4.8	0.439
IgE	108.9 ± 15.7	172.30 ± 24.8	108.90 ± 51.4	0.096

Significant difference (*p*<0.05) by age and sex-adjusted ANCOVA among the values of clinical parameters by degree of exercise. *p<0.01 vs exercise (–) group and ^#^*p*<0.05 vs exercise degree (+) group.

**Table 3:** Odds of degree of exercise according to arginase activity

Explanatory variable	Arginase activity				*p* trend
	Q1	Q2	Q3	Q4	
Model 1	1	1.99 (0.77–5.17)	2.41 (0.94–6.19)	**4.11 (1.62–10.42)**	**0.003**
Model 2	1	1.49 (0.54–4.12)	1.84 (0.68–4.97)	**2.91 (1.04–8.14)**	**0.035**
Model 3	1	1.38 (0.47–4.04)	1.78 (0.61–5.16)	**3.77 (1.24–11.46)**	**0.016**

Model 1: Odds of degree of exercise according to arginase activity with no adjustment. Model 2: Odds of degree of exercise according to arginase activity, adjusted with age and sex. Model 3: Odds of degree of exercise according to arginase activity, adjusted with age, sex, BMI, l-arginine, l-citrulline, l-ornithine, hs-CRP, FENO, NOx, FEV1%, IgE, HDL-c, cigarette smoking, and alcohol intake.

**Table 4:** Changes in nitric oxide-related parameters before and after chronic exercise training for one month

Variables	Total		*p*	Male		*p*	Female		*p*	Age ≤34		*p*	Age ≥35		*p*
	(*n* = 41)			(*n* = 19)			(*n* = 22)			(*n* = 22)			(*n* = 19)		
	Pre-exercise (*n* = 19)	Post-exercise (*n* = 21)		Pre-exercise	Post-exercise		Pre-exercise	Post-exercise		Pre-exercise	Post-exercise		Pre-exercise	Post-exercise	
Age	34.24 ± 12.0			34.68 ± 12.34			33.86 ± 11.97			24.27 ± 4.01			44.55 ± 8.22		
Sex (M/F)	19/22									10/12			9/10		
Weight	62.1 ± 1.78	61.8 ± 1.8	0.842	69.6 ± 2.4	69.2 ± 2.4	0.793	55.5 ± 1.6	55.4 ± 1.6	0.940	62.5 ± 2.2	62.1 ± 2.2	0.769	61.6 ± 2.8	61.4 ± 2.9	0.964
Arginase I	4.8 ± 2.6	4.7 ± 2.5	0.766	4.8 ± 1.9	4.2 ± 1.4	0.290	4.9 ± 3.1	5.1 ± 3.2	0.835	4.7 ± 2.4	4.1 ± 1.7	0.252	4.9 ± 2.9	5.4 ± 3.1	0.609
l-Arginine	156.1 ± 35.4	138.3 ± 31.3	**0.001**	165.2 ± 35.3	146.7 ± 5.5	**0.040**	148.3 ± 34.4	131.0 ± 35.3	**0.012**	148.7 ± 27.8	134.6 ± 30.4	**0.024**	164.8 ± 41.8	142.4 ± 32.5	**0.019**
l-Citrulline	32.8 ± 8.0	34.6 ± 6.8	0.168	33.7 ± 7.6	35.5 ± 7.1	0.361	32.1 ± 8.3	33.9 ± 6.6	0.318	32.6 ± 8.4	35.0 ± 8.3	0.266	33.1 ± 7.6	34.2 ± 4.7	0.424
l-Ornithine	72.8 ± 32.7	84.30 ± 33.7	0.101	68.30 ± 30.2	87.5 ± 33.1	0.103	76.7 ± 34.9	81.6 ± 34.6	0.566	79.1 ± 4.0	89.2 ± 3.5	0.367	65.6 ± 19.4	78.7 ± 32.2	0.112
NO_2_	141.9 ± 45.5	160.4 ± 53.4	**0.039**	121.9 ± 24.8	145.2 ± 44.1	**0.043**	159.21 ± 52.4	174 ± 58.2	0.298	150.3 ± 54.9	169.0 ± 60.2	0.180	132 ± 30.1	150.5 ± 43.9	0.107
NOx (NO_2_^−^ + NO_3_^−^)	34.3 ± 20.2	33.7 ± 21.0	0.883	34.80 ± 20.4	29.4 ± 13.8	0.323	33.8 ± 20.7	37.3 ± 25.4	0.611	31.2 ± 16.4	30.5 ± 13.7	0.863	37.9 ± 24.0	37.3 ± 27.1	0.948
SBP	130.4 ± 15.2	128.2 ± 15.3	0.115	137.1 ± 15.5	136.9 ± 12.4	0.944	124.7 ± 12.6	120.6 ± 13.6	**0.030**	126.5 ± 10.4	127.1 ± 11.5	0.700	135.0 ± 18.6	129.4 ± 19.1	**0.026**
DBP	79.2 ± 12.0	76.70 ± 11.7	**0.032**	83.6 ± 12.8	80.7 ± 11.2	0.125	75.4 ± 9.9	73.2 ± 11.3	0.150	73.3 ± 8.4	74.60 ± 9.4	0.631	83.7 ± 13.9	79.1 ± 13.9	**0.019**
Urea	30.20 ± 9.3	39.40 ± 14.3	**<0.001**	31.1 ± 9.9	33.8 ± 9.2	0.435	29.4 ± 8.9	44.3 ± 16.3	**<0.001**	32.1 ± 9.1	45.40 ± 15.7	**0.002**	28.1 ± 9.3	32.6 ± 8.8	0.101

The values represent the mean ± SEM, and bold represents statistical significance.

**Table 5:** Changes in amino acid ratios between before and after chronic exercise

Variables	Mean ± SEM (*n* = 41)		*p*
	Pre-intervention	Post-intervention	
Arg/(Cit + Orn)	1.59 ± 0.56	1.22 ± 0.33	**<0.0001**
Arg/Cit	4.88 ± 1.06	4.07 ± 0.95	**<0.0001**
Arg/Orn	2.61 ± 1.39	1.85 ± 0.75	**<0.001**
Cit/Orn	0.55 ± 0.30	0.47 ± 0.20	0.111

Bold indicates statistical significance.

**Table 6:** Primer sets for real time PCR

Target gene	Forward primer (5' to 3')	Reverese primer (5' to 3')	Source
SOD1	CTAGCGAGTTATGGCGACGA	TAATGCTTCCCCACACCTTC	qPrimerDepot
SOD2	GGAGAAGTACCAGGAGGCGT	TAGGGCTGAGGTTTGTCCAG	qPrimerDepot
Catalase	TGGGATCTCGTTGGAAATAACAC	TCAGGACGTAGGCTCCAGAAG	PrimerBank
GPX1	CAACCAGTTTGGGCATCAG	AAGAGCATGAAGTTGGGCTC	qPrimerDepot
SIRT3	ACCCAGTGGCATTCCAGAC	GGCTTGGGGTTGTGAAAGAAG	PrimerBank
